# Review of the Gibraltar Fever

**Published:** 1830-10-01

**Authors:** 


### Review of the Facts and Arguments brought forward by Dr. Barry, at the Royal College of Physicians, relative to the late Epidemic Fever in the Fortress of Gibraltar. By *Hugh Fraser*, Esq. Surgeon of the Civil Hospital, and late Assistant Surgeon, 12th Regiment.

The dreadful ravages which the celebrated Fortress of Gibraltar has sustained, during the present century, from the invisible hand of pestilence, have called forth the sympathies of the British nation—and excited the attention of the medical profession. Deeply do we lament that, in investigations bearing on the health and lives of our fellow creatures, the medical spectators should exhibit such a melancholy diversity, not only of opinions, but of facts—and still more that they should have either cause or inclination to question, nay, directly to impugn, the veracity of each other! No investigation has ever given rise to such acrimony in discussion as that of "*contagion and non-contagion!*" We should be sorry to see the tremendous conflicts of Bancroft and Chisholm renewed, in the persons of Barry and Fraser. We certainly think that several of the expressions in Mr. Fraser's paper are too acrimonious; and that his facts and arguments would have suffered nothing, but rather gained in strength, by milder language. Nevertheless, we have not refused to give publicity to this document, because private feelings must give way to the public good. The question of importation or domestic origin, as respects the Gibraltar fever, is one of immense interest, and deserves a philosophic investigation. This inquiry has been opened in France—and now in England. Our own impression was stated while giving an *exposé* of Dr. Barry's paper, read before the College of Physicians—and all that we have since learnt on the subject has only tended to strengthen our conviction, that the Gibraltar fever was just as much imported from the Havannah with a cargo of cigars, as from old England, in the snuff-boxes of Drs. Pym and Barry.

The summary which we gave of Dr. Barry's paper excited the attention of Mr. Fraser, and, apparently, gave rise to the present series of strictures. We shall, according to our custom, condense and quote, as we deem advantageous to the author and the public.—The following declaration is absolutely necessary.

"If, in the course of my remarks, language or expressions drop from me which may appear strong or uncourteous, I shall be sorry; as I disavow all personal animosity or hostile feeling towards Dr. Barry. But, as he has so mis-stated facts, and so darkened many of the most important truths relative to the history of the epidemic in question, that I fear it will be impossible to enter into an analysis of his paper without the appearance of personality, I shall only observe, once for all, that my object is truth; and if *this* is not to be found (as some have stated) in the arguments either for or against contagion, to whom are we indebted for so grave a charge? Certainly not to the non-contagionist. Before the arrival of Dr. Pym in the garrison, towards the termination of the malady, the word contagion had nearly become obsolete:—So clear and satisfactory were the proofs of the endemic origin of the disease, to those who had witnessed its rise and progress, that no one doubted the accuracy of this doctrine; and Dr. Barry himself, soon after his arrival, drew up

Reel : 8/- 36 - 6

a paper setting forth a variety of arguments corroborative of the same—but more of this hereafter." 2.

Mr. Fraser then proceeds to examine, seriatim, the postulates of Dr. Barry, as given in our 24th Number, p. 539 *et seq.*

"POSTULATE 1.—The late Gibraltar epidemic (says Dr. Barry) was a fever of one paroxysm—cold—hot—sweating. If slight, nothing more—if severe, yellowness about the third day, black vomit, hiccup, total suppression of urine, copious hæmorrhage of black blood from the nose, gums, anus, vagina, and death from 4th to 6th or 7th day, always without fever."—*Med.-Chir. Rev. No.* XXIV. *p.* 539.

"If this expression of 'PAROXYSM' be not meant to convey a 'sophism,' it is, to say the least, obscure. If the term be here intended to convey a notion, that during the febrile act there were no remissions or exacerbations, the definition is most improper. Having been present during the whole of the epidemic, my opportunities of examining the disease have been more extensive than those of Dr. Barry, who only arrived at its decline, and the opinions I have formed are, therefore, necessarily grounded on a larger experience; for I must, at least, have seen and treated twenty cases of the disease for every one that Dr. Barry saw or treated; and I assert that remissions, more particularly in the milder cases, were observed, and sufficiently marked to warrant me to pronounce the definition given to be based in ignorance or in error.

"To give a definition of the fever that might answer the purposes of nosology would be extremely difficult; because it presented itself in as many variegated forms as the shades of the rainbow:—and had Dr. Barry wished to have given a more correct view of it than he has done, he would have been nearer the truth, had he said that it was a fever of a 'continued form,' attended with remarkable 'lulls' and 'exacerbations,' without the latter being preceded by any very regular or well-marked rigor—still, however, in a vast number of the cases, with a sensation of chill sufficiently apparent to mark the commencement of a primary or temporary morbid association. Is it likely that a fever which, in a great proportion of the cases, lasted for ten or twelve days, and which occasionally ran on to the 15th or 20th day, could consist of one paroxysm? No; but such a definition answers the views of the Doctor, in his endeavour to prove this a fever 'sui generis,' and different from our sporadics, or the marsh remittent of the adjacent coasts.

"Whether our epidemic radically belongs to this latter form of disease, is not necessary for me to discuss;—all I wish to insist upon is, that most decided 'remissions' and 'exacerbations' took place, unless, perhaps, in fatal cases, where the impetuosity of the onset was often such as to extinguish life before re-action could be induced; and, even when induced, would rush rapidly on to black vomit and dissolution, without giving the physician a chance, as Dr. Cullen would have said, of 'obviating the tendency to death.' In such cases, renewed paroxysms were not to be expected; but, in the cases of recovery, most perfect remissions took place, as may be proved from every medical register kept in the garrison.

"Dr. Amiel, who has witnessed four epidemics here, and whose accuracy of observation is so well known, gives the following answer to query 14th from the Army Medical Board.

'I believe the epidemic differs only in degrees of intensity from the Autumnal bilious remittent fever of this climate; and I am the more strongly induced to adopt this opinion, as I have occasionally witnessed, in times when the bilious remittents were frequent, cases attended with the violent attack, the high malignity, and the rapid dissolution, which more generally mark the character of the epidemic;—and, during the epidemic season, instances have been frequent in which the disease has retained the mild form and the distinct character of the bilious remittent.* This was particularly remarked in 1810, and during the epidemic of 1814; and has also

* Dr. Arejula, in his 'SUCCINCTA DESCRIPTIO FEBRIS EPIDEMICÆ,' says, 'that in 1803 the febrile actions frequently assumed a remittent type by cold, hot, and sweating stages, being evidently distinct, and the disease was frequently protracted to the 11th, and sometimes to the 14th day.'

been remarked at the breaking out of every epidemic, probably from the circumstance of the morbific causes not having yet acquired their concentrated energy and virulence.'

"The disease has no pathognomonic symptoms. Yellowness was not present in about one-third of the patients, and was often wanting when death took place. The black vomit was entirely confined to the mortal cases. Suppression of urine was mortal—retention, not so. Hæmorrhages were not mortal;—on the contrary, they appeared to me, in very many instances, as preludes to a healthy movement. What Dr. Barry wishes to convey, by saying that 'death took place always without fever,' unless in the last stage of the paroxysm, I do not understand;—but it is not necessary here to enter farther into the subject; and, passing over the 'anatomical characters' given in the 2d postulate, which I mean to advert to by and bye, I shall proceed to the consideration of the others."

'Postulates 3d, 4th, and 5th.—Gibraltar is certainly one of the cleanest towns in Europe, not even excepting Bath—well supplied with the best fresh provisions, vegetables, &c. no poor; not a single beggar.'

'4th. The population 21,000 at the commencement of the epidemic, spread over a surface three miles long by more than one quarter broad; besides the bay, the Neutral Ground, and Catalan Village.'

'5th. No town nor territory in Europe has been more improved within the last ten years.'

"I believe that every one who knows any thing of Gibraltar, will acknowledge the improvements made in the Garrison, under the paternal government of Sir George Don, during the last ten years, to have been very extensive; but, as to its being one of the 'cleanest towns in Europe,' 'not excepting Bath,' this is an assertion not borne out by the facts, and could only be made by a person unacquainted with the habits of the people of Gibraltar. But why argue the subject? Let any one attentively peruse the following letters of the late lamented Dr. Hennen, and then deny that Gibraltar abounds in fever-exciting causes, and I will yield to the Doctor's dogmas.

'*Inspector's Office, Gibraltar*, 29*th Aug*. 1828.

Sir,

I have the honor to state, for Your Excellency's information, that, within the present week, I have personally visited, or received reports of, five cases of the '*bilious autumnal remittent fever*,' which has made its appearance in a neighbourhood at the back of Hargrave's Parade. One of the cases which I saw had assumed all the appearance of yellow fever, and proved fatal yesterday, and two of the cases reported to me are stated to be of a similar nature, and will probably lead to a similar event.

Independent of these cases, which occurred among the poorer classes of inhabitants, three or four of the family of Mr. Martin, chief clerk in the Civil Secretary's Office, have been attended in a similar disease by Mr. Hugh Fraser, Surgeon of the Civil Hospital; and it is not a little remarkable, that the fatal case seen by me, occurred in a woman who was a servant of Mr. Martin's.

I shall lose no time in making a most minute personal examination of the whole of this neighbourhood, the result of which I shall duly report to Your Excellency. In the mean time, I have taken upon myself to order Mr. Beatty, the Director of the Scavengering Department, to take instant measures for cleansing the drain, by throwing a sufficient quantity of water down the grating above alluded to, and then covering it, so as to prevent the emanation of all foul vapours. I have further directed him to survey the drain throughout the whole course, to detect, if possible, any obstruction that may have occurred.

I shall order the medical officer in charge of the district to be most vigilant in the performance of his duty; and I beg to request that Your Excellency will urge the inspectors of districts to a frequent and accurate performance of the duties allotted to them. I would also beg Your Excellency to issue orders to the police serjeants to make the most minute and rigorous inspection of every part of their districts, as I have every reason to believe, that one of the cases alluded to in the first paragraph of this letter was ill from Saturday to Thursday, before the circumstance was reported. It is, however, but proper to say, that the patient had shut herself up, and that the house in which she was had every appearance of being untenanted.

In concluding, it is proper for me to acknowledge to Your Excellency the cordial co-operation which I have experienced from Messrs. Fraser and Wilson, of the Civil Hospital, and from Messrs. Mathias, Dias, Ming, and Lopez, civil practitioners, who lost no time in directing my attention to every particular that might affect the public health, that came within their observation.

I have the honor to be, &c.

(Signed) J. HENNEN, M.D.

*Inspector of Hospitals.*'

*To His Excellency the Governor,*
*&c. &c. &c.*

---

'*Inspector's Office, Gibraltar,*
*29th August*—*9 o'clock, p.m.*

Sir,

In reference to my letter of this day's date, I have now the honor to inform Your Excellency that I have minutely inspected District No. 24, in company with Mr. Wilson, of the Civil Hospital, Mr. Woods, the medical officer attached to that district, and other staff officers, '*and it is with much regret I have to state to Your Excellency, that in almost every step I took in that district I had reason for surprise, not that the fever had broken out there, but that it had not extended further.*'

It would lead me into matters not immediately connected with professional points, were I to enlarge upon all that I have seen and heard during my inspection of that district; but the conclusions to which I am '*irresistibly led*' are as follows;—and I beg to claim Your Excellency's special attention to them, as the only means of preventing a repetition of those horrors which occurred in this garrison, at the period of Your Excellency's happy arrival in 1814.

1st. From whatever causes it may have proceeded, the '*pauper population* is *dense to a degree*, '*incredible except by those who have seen it.* In sheds,' *without ventilation*, '*without drainage*,' and generally composed of the slightest materials, *in tiers of beds, as close as a crowded transport, numerous individuals sleep.* They go out to their work at an early hour, and return at gun fire, locking up their miserable places of nocturnal shelter during the day, and leaving them saturated with the steams of their bedding, their food, and the overflowing receptacles of their ordure. The detail would be too disgusting to be entered upon; but I most respectfully submit to Your Excellency the indispensible necessity of sweeping away the whole of those sheds, which I have every reason to suppose are unauthorized by the Government, and are solely the offspring of the most sordid avarice. So confident have been the owners of these sheds as to their permanence, that some of them are actually covered with sheet iron—a measure which, while it may tend to put money into the pockets of the owner, by preserving the wretched sheds, must especially conduce to render these places hot-houses of contagion.

Might I presume to offer my opinion, a committee of civilians, military and medical officers, should be immediately appointed, to enquire into the state of all the temporary buildings throughout the garrison, and if they are deemed incompetent for the purposes of human accommodation, and with a risk to public health, that they may be forthwith razed to the ground, as I understand many of them have heretofore been, although subsequently, from *increased demands* 'on the part of the *lower orders*' for *shelter*, they have sprung up with *incredible rapidity*, contrary to the spirit of those admirable police regulations which were laid down by Your Excellency.

Although my recent observations have led me to a positive conclusion with regard to the state of District No. 24, I cannot doubt that all the other districts are comparatively in a similar state. 2d. Without wishing to implicate any individual in a charge of neglect of duty, I would suggest that the police serjeants of districts should receive the most positive orders to attend especially to the duties connected with the cleanliness of the places committed to their care. On this subject, I shall only say that a most respectable medical officer has stated to me, that he '*rarely sees a police serjeant perambulating his bounds*;' and, in truth, the veracity of his assertion was confirmed to me by *more senses than one.* Under this head, I may mention that I had repeated evidence that Your Excellency's orders, with regard to placing barrels, buckets, &c. for the reception of dirt in commodious situations for removal by the scavengers, appear in many cases to have been altogether neglected.

I shall feel it both a duty and a pleasure to accompany any committee that Your Excellency may think proper to appoint, to examine into the *multiplied causes which at present threaten the public health.* I shall lose no opportunity of visiting every part of this garrison, and shall report to Your Excellency every circumstance which, in my opinion, may tend to the preservation of the public health.

In consequence of Your Excellency's private note of this day's date (of 3 o'clock) I have had the drains alluded to opened up, washed down, and impregnated with quick lime; but all these measures must fail, if the spirit of Your Excellency's General Police Orders are not strictly and conscientiously attended to.

I have ordered the medical officers, of all ranks, to be upon the alert on this occasion, and I shall endeavour to second my orders by my example, and

I have the honor to be, &c.

(Signed) J. Hennen, M.D.

*Inspector of Hospitals.*'

*To His Excellency the Governor,*
*&c. &c. &c.*

That the accumulation of animal and vegetable corruption within the garrison, during the year, 1828, was immense, Mr. Fraser thinks will be admitted by every one acquainted with the place. He says it is an incontrovertible fact, that the drains generally, for some months prior to the breaking out of the fever, were loaded with putrid matters—many of them being obstructed—some of them burst. The noxious vapour exhaling from them, he observes, was, at times, so powerful as to produce *vertigo and vomiting* in several individuals. He does not wish, however, to infer that the exhalations from these drains were the sole cause of the fever—he only means to shew, that decomposed animal and vegetable matters were in greater abundance in 1828 than in many preceding years. In respect to Dr. Barry's averment, that "there are no poor, not a single beggar" in Gibraltar, Mr. Fraser offers the following observations.

"It is true, a person is seldom seen in rags, in the public streets, openly asking for charity; but the misery of many individuals is not the less on that account; and I know that wretchedness and poverty prevail here, to a degree equal to that of any other town having the same number of inhabitants. Begging in the streets is forbidden; but the following answer of Mr. White, Collector of Revenues, to the Board of Commissioners, at its 21st sitting, 16th March, 1829, will show the condition of the lower orders.

Question:—Have you found in your experience, as Collector of the Crown Rents, cases of deep distress and penury amongst the lower order, who were tenants of the crown immediately preceding the epidemic? and have you any reason to know, from any other means open to you, that similar cases of penury and distress were numerous in the garrison, amongst the foreign population or natives, in 1828, previously to the epidemic?

Answer:—I have. From the time that I came here, in August, 1827, it has been difficult to collect the rents of the small renters. This difficulty became each month greater, until a few months previously to the epidemic, to get any rent from the small renters without bringing them before a magistrate;—and even then I often got nothing, for there was nothing whereon to levy distress. I have further to state, relative to the distress of these small renters, that I have, several times, caused their dwellings to be visited, when they were always reported to me to be in a wretched state, by Mr. Woodward and different other people. One particular room was reported to me by Mr. Woodward, as containing a family of ten persons, who, if stretched on the floor, could not, calculating according to, or by, Mr. Woodward's measurement, have more than four inches between each two persons. Many of the King's rooms were also found, at night, to be occupied by *beggars*, and people of other descriptions, who, at the dawn of day, disappeared, and could not be found during the day." 14.

M. Fraser next proceeds to examine the sixth and seventh postulates of Dr. Barry, which stand as follow:—

6. "Nine persons were sick of '*some fever*,' and two died on board a ship from the Havannah, on her passage to Gibraltar, in the Summer of 1828—whilst this ship lay in quarantine from 27th June to August; three more were ill, and one of these was received into the Civil Hospital on 6th August, as '*febris intermittens*,' but he had no paroxysm after admission.

7. The two first persons taken ill, were the son and daughter of a bomb-boat man; they had been on board some ship shortly before. One died on the 17th, the other on the 20th August; both yellow, both with dark vomiting. The family of the health guard of this ship was attacked on the 21st August. A servant-maid in the Civil Hospital on the 23d August. The one military case was on 2d September. This man lodged between the two houses first attacked."—BARRY.

In reference to the first part of Postulate VI. Mr. F. remarks thus:—

"Suppose one thousand persons had been sick of '*some fever*,' as Dr. Barry calls it, on board a ship from the Havannah, and that the whole had died—what does it prove? Nothing; unless that *same fever* can be incontrovertibly shown to have been contagious, and to have prevailed in the Havannah at the time the ship sailed, and to have been introduced into this garrison from the ship, by communication or contact of infected persons or goods. I say it proves nothing.

It would lead me far beyond the limits I have prescribed to this paper, were I to enter into the detail of all the means practised to fix the blame on this ship. Suffice it to say, that the Board of Commission already alluded to, after wading through a mass of evidence, and listening to the stories of witnesses got up for the occasion, came to the conclusion (the majority at least) that there was not the slightest proof for referring the introduction of our epidemic to the ship DYGDEN, or to any other vessel. I subjoin the opinions of Colonel Chapman, Civil Secretary, and of Judge Howell, both of whom may be supposed capable of weighing evidence as fairly as any other of the members of this Board. They are as follows:

*Colonel Chapman.* "Judging from the evidence produced before the Board—the manner in which it has been given, together with the description of persons who have been brought forward as witnesses, I am decidedly of opinion, that the late epidemic disease is of local origin. As to the importation of the late epidemic, I am of opinion that the attempt to prove the introduction of the disease, after months of previous enquiry by those who wish to prove it, have totally failed."*

*Judge Howell.* "Upon a careful review of all the proceedings before this Board, I am of opinion that the evidence brought forward has totally failed to prove that the late epidemic disease was introduced from any foreign source, either by the Sweedish ship DYGDEN, or by any other means; and I am farther of opinion that the late epidemic had its origin in Gibraltar."*

As to the three sailors said to have been taken ill whilst the Dygden lay in quarantine, one of which was received into the Civil Hospital as "febris intermittens,"† the following answers given Mr. Fraser himself to the Board of Commissioners, are set forth in opposition to Dr. Barry.

* Vide Board of Commission, 46 sitting.

† Why Dr. Barry alludes only to '*one man*,' when he knows there were '*two*'

Question. "Do you recollect two men belonging to the Dygden having been admitted into the Civil Hospital? When were they admitted, and with what complaints?"

Answer. "I do. John Glisson was admitted into the Hospital on the morning of the 7th August, and William Barrow on the evening of the same day. I have since understood that these two men were discharged from the Dygden, the day previous. John Glisson had a *contusion* of the arm. William Barrow was admitted under the head of '*intermitting fever;*' but showed no symptoms of fever, during his stay in the hospital; and was discharged three days afterwards. He appeared to labour under a chronic affection of the liver, or HEPATITIS. He was much wasted in muscular strength, and had a dark marasmal appearance."

Question. "Did you enquire into the previous history of this man's disease, and did you take any notes of it?"

Answer. "I noted the following in the Medical Register, viz. 'Admitted from the Havannah, where he has had fever.' I think these are the words noted in my Register."*

As to the third case of sickness on board the Dygden, Mr. F. thinks that Dr. Barry must allude to the mate of that ship, for whom some medicine was procured at the hospital by one of the merchants of the rock, who merely stated that the man had head-ache, and was costive.

"Such is the front and bearing of one channel on which Dr. Barry rests his proofs of the introduction of a contagious distemper into our garrison—one man '*said*' to have had '*ague*,' at the Havannah—a second, a '*contusion of the arm*'—and a third '*constipated bowels!!*' Now, let us see on what grounds the Doctor rests his other proofs of introduction; and first, for the bomb-boat man, and his crew. The Doctor here, with his usual tact, and as if not satisfied with sending one or two men to the Civil Hospital from the unfortunate Dygden, labours hard to establish a more direct line of introduction, and tells a plausible story enough of the communication in which this bomb-boat man, his two children, and a boy, had with the ship; and says that they all sickened soon after being on board, and that three out of four died. This is all very pretty; and, on paper, looks well; but let us examine a little more particularly into the real facts of the case, and then the unbiassed can judge what degree of credit is to be given to this part of the Doctor's statement.

The whole story hinges on the evidence of a boy named Caffiero, not thirteen years of age, and a few washerwomen:—and, were it not trifling with the time of the reader, as well as my own, I could here recapitulate the whole evidence as given by these worthies before the Board of Commission: but it is so truly contemptible, and so much at variance with truth, that it disgusted every honest man who heard it. I shall pass it over '*sub silentio*,' and content myself by quoting the declaration of the bomb-boat man's wife, named Fani, as given before a competent authority, and who is ready to confirm it on oath if necessary. I shall also quote her answers before the Board on the same subject, which are of similar import.

*Gibraltar*, 14*th November*, 1829.

Catalina Fani, widow of Felix Fani, (alias Mabla) has this day made a statement in presence of the undersigned to the following effect, and which, if required, she states her readiness to corroborate on oath, before competent authority.

Declares herself to be the widow of Felix Fani, who died in the Autumn of last

---

admitted into the hospital, I am at a loss to know; unless the one '*febris intermittens*' suited the Doctor's purposes better than the other, which was a case of '*contusion*.'

* Vide Board of Commissioners, 37 sitting, 19th April, 1829.

year. Says that her husband was about 68 *years of age*, and of a *broken constitution*, that he had resided in Gibraltar about 33 or 34 years.

States that in 1804 she was the wife of a man named Salvador de Ortega, who was then the intimate friend of Felix Fani, the person above alluded to, and to whom she was married in 1805, after the death of her first husband (Salvador de Ortega,) who died of the epidemic fever in 1804.

States and is ready to make oath, that her second husband (Felix Fani) had in 1804, a severe attack of the epidemic fever* of that year; and that she, and her first husband (Ortega), visited him frequently. That in fact, she herself attended him jointly with another woman since dead. That during his illness, his residence was in a small house at the bottom of the stairs in front of the Civil Hospital, then called the Blue Barracks. That during the epidemics of 1813 and 1814 her husband resided in Gibraltar without experiencing any indisposition from the fever of those years.

States that after the death of their two children, (Salvador and Catalina Fani) mentioned in the proceedings of the Board of Commissioners to have taken place about the middle of August 1828, her husband became greatly distressed in mind; that, on a certain Thursday, the police came to their house, to warn them, in common with others of the district, to remove into camp. That on that day, her husband particularly complained of a large rupture which he had had for many years, and which occasionally troubled him, being worse than usual, and increased in bulk. That she had her husband brought to the Civil Hospital next day, where he was seen by Mr. Fraser, and others: but not being a fever case, he was sent back to his house. On the evening of the next day, they were, like all others in her district, sent to camp on the Neutral Ground.

In camp, her husband was seen by Dr. Hennen, and others; but was not ordered to the Lazaretto, to which all fever patients were sent. He died in their tent, on Monday morning—the tent was not ordered to be fumigated or washed by any one, and that she took no precautions to prevent contagion, as she was sure her husband did not die of the fever. Declares that there was no vomiting, no yellowness of skin, or other usual signs of a bad fever. Says that he complained greatly of a pain in his throat, and that it was a question among the medical men who saw him, as to bleeding him; but that they said they had no lancets with them.

With reference to her husband having gone on board any ship in the bay, at any time last summer, she most positively asserts that it was not so, as he never went out any where (he being an old infirm man) without telling her. Is sure that he had not been in a boat for ten years past.

She is equally certain that her two children (Salvador and Catalina Fani) were not on board any ship or boat, as stated in the proceedings of the Board of Commissioners, as they were always under her eye, and were not running about like other children, or ever in the habit of going on the water.

States that, with respect to the boy Francisco Caffiero, neither she herself nor any of her family knew any thing about him, and that what he stated to the Commission, as to her boy Salvador, (13 years of age,) and Catalina, (11 years of age,) having gone on board of ship, is '*a made up falsehood*.'

States that her children had not any of the symptoms of the epidemics of Gibraltar, with which she has been familiar during her long residence. That the Doctor (Lopez) observed some indigested portions of figs in what they passed by stool. That they had no vomiting. That their appearance after death was not changed like that of those persons whom she has seen die of the yellow fever, and that she cannot make herself believe that they died of that disease.

Says that during the illness of the children five of their play-mates (the children of her gossip Joaquina) constantly came to see them, but that none of these children were taken ill. That the whole of these children, however, passed the epi-

---

* According to the doctrine of Dr. Pym, this man was impregnable to a second attack, and could not have had the disease in the epidemic of 1828; although the contrary will be seen to be inferred by Dr. Barry.

demic afterwards, when permission was given to some of the families to return to their expurgated habitations.

(Signed) A. Browne, M.D.
Assist. Surg. 23d R.W.F.
Hugh Fraser,
Surg. of the Civil Hospital.
J. Gillkrest, M.D.
Surgeon 43d Regiment.

I, the undersigned, do hereby certify, that on this thirteenth day of May, one thousand eight hundred and thirty, Catalina Fani, in the foregoing statement named, appeared before me, and I then interpreted to her the whole of the said statement, which she confirmed in every particular, in the fullest and most positive terms, and added thereto, that her late husband had, for several years previous to his death, left off going at all into the Bay, and that, being an old and infirm man, he gained his livelihood by purchasing tobacco and making it into cigars.

(Signed) Alexander Shea,
Not. Pub. Gibraltar.

This declaration, Mr. Fraser thinks, must negative the idea that any of the four persons in question had gone on board the Dryden, or into the bay—or that Felix Fani died of the epidemic, since the man was destroyed by *strangulated hernia.* It is very doubtful of what disease the children died; but Mr. F. thinks it proved beyond all question that they did not die "with yellow skin and black vomit," as asserted by Dr. Barry.

Mr. Fraser next proceeds to the case of his own servant maid, who is stated by Dr. Barry to have been attacked with the fever on the 23d of August.

"This woman (a Portuguese) had been in my service three years. On the *third* of August, 1828, she had an attack of fever, which endured with severity for ten or twelve days—her skin became yellow, and she suffered from irritability of the stomach. I considered her disease at the time, in common with Mr. Peter Wilson, late of this Hospital, to have been one of those sporadical attacks of yellow fever, which every now and then appear here. I stated my belief at the time to several members of my family, that her disease probably arose from the proximation of an extremely foul drain to the window of her bed room. On the 2d September, Dr. Hennen, in company with other medical officers, visited this hospital; and, in conversation, I remarked, that we ought to be extremely circumspect in giving farther alarm of fever, for that, in the event of its subsiding, the public might be inclined to attach censure to the energetic measures which he (Dr. Hennen) was adopting with the view of suppressing the malady; at the same time, mentioning the case of my servant maid, as also the servant of Mr. Hassan, Keeper of the Civil Prison, who had been similarly attacked, and who lived in District No. 5. Dr. Hennen noted these two cases at the time, and directed his clerk, who was present, to have them appear in the sick report of that morning, which they do; and opposite to my servant it is thus written—"Servant to Mr. Fraser, who has been ill more than a week;" clearly showing the inaccuracy of referring the date of attack to the time of being reported, as the insertion of the cases into the morning state, was merely intended by Dr. Hennen to shew all the cases of fever which had come to his notice during the month, and the remark of the clerk, had been made of his own accord, without knowing any thing of the particulars of the case. This fact I more than once mentioned to Dr. Barry; so that it is not in ignorance that it is brought forward in support of his favorite doctrine. The obliquity of the Doctor's intentions is indeed very apparent here:—observe how studiously he keeps out of view the servant at the Civil Prison, though appearing in the same report. But here there was a link wanting in the Doctor's chain: no contact with infected persons or things could be shown—neither John Glisson, or William Barrow of the Dygden, or any of the bomb-boat man's crew, had been near the Prison.

In respect to the first military case of fever that occurred, on the 2d of September, Serjeant Oldroyd, Mr. Fraser avers that Dr. Barry is incorrect in making him a lodger in district 24, "between the two houses first attacked." The man was not married, and had no permission to lodge or sleep out of the barracks. It has been stated, indeed, that this man kept a woman in the above district—and if so, Mr. F. does not wonder at his imbibing the febrific fumes of that foul locality during his visits to his paramour. Mr. Amiel, however, surgeon to the regiment, in his examination before the Board, at its 17th sitting, (7th March,) observes that Oldroyd "was found drunk and disorderly under the bridge of the Naval Hospital, two days before he was taken to the hospital, i. e. 31st of August," a place which afterwards proved a hot-bed of fever, and where he thinks the serjeant was just as likely to have contracted his fever as in district No. 24.

"Postulate 8.—This disease left upwards of 5000 persons untouched, who had passed it before. There is not a single authenticated case of second attack in Gibraltar.

Of 164 military orderlies in the regimental hospital 141 caught the disease. Of 61 civil attendants who voluntered to attend the military sick (being largely paid) two only were attacked, and these were the only persons of the 61, who had not passed the disease in some former epidemic. This fact is taken from the official returns of regimental surgeons."

The doctrine of 'non-liability to a second attack,' as it now obtains in Gibraltar, is an imposing argument, more specious however than real; it is obviously one open to much fallacy; for the proposition is more or less assumed. Have the 6000 persons alluded to been all interrogated, as to the nature of their first attack? Have none of them left the garrison for other countries and returned? has it been ascertained that all these people lived within the circle of epidemic influence in 1828, and if so, is it certain that none of them sickened to a greater or lesser degree? did none pass through a sub-acute form of the malady? Knowing that none of these interrogations can be answered in the affirmative, I hold it to be ridiculous to argue further; for no conclusions could be drawn that would not be open to most serious objections. The experience of another epidemic, or more, will be hardly sufficient to place this question on such a basis as to be useful to the profession. It must, however, be acknowledged to be a belief, at present held by the natives of Gibraltar and of Spain, paramount to all others, except their conviction in the truth of the importation of the disease; but it does not follow they are correct. The history of man, in every age, shows him prone to embrace notions, however false, which pamper his hopes, and allay his fears; and to tell any of these natives, that a first attack of yellow fever was not to exempt them from a second, would be received with the same repugnance, as the telling an old and pious lady on her death bed, that men of erudition had doubted the existence of her soul. That a belief so rivetted, has the most salutary effect in resisting a second attack, no one can deny; for it renders the system impregnable to the impression of fear, which all who have witnessed an epidemic of yellow fever, will admit to have a powerful influence in keeping up and extending the disease. But, as the object of this paper is not the discussion of speculative points, I shall proceed to the examination of the proofs on which the immunity of these 5000 people is said to rest.

At the time that the Board of Commission sat here, a Board of Medical Officers was ordered to investigate this subject. It was composed of the French commission, Drs. Chervin, Louis, and Trousseau—Dr. Barry and myself—besides some half-dozen Spanish doctors; but the principles on which it was conducted, were, to me, as well as to Dr. Chervin, so objectionable that I addressed a letter to His Excellency the Governor, expressive of my sentiments on the subject, and refusing to sign its proceedings. The letter is as follows;—and shows, in a tolerably clear manner, the spirit which influenced this board.*

* I regret not being able to give a copy of the letter of Dr. Chervin refusing to sign the proceedings, and containing his dissentient opinions, which he also addressed to His Excellency the Governor on this occasion.

*Gibraltar, 1st April*, 1829.

Sir,

In reference to the Board of Medical Officers assembled at your Excellency's order, in the chronicle of the 24th January last, to examine into the liability or non-liability of a second attack of the epidemic yellow fever which prevailed here last year, and of which Dr. Pym did me the honor to be associated a member, I beg to remark to your Excellency, that, at a meeting held yesterday, for the purpose of concluding and signing the report to be made to Your Excellency on this most important question, I felt myself reluctantly obliged to dissent from the conclusion therein given; and, it is with much concern, I have to state to Your Excellency, that when this report shall be laid before Your Excellency, my signature will be found to be wanting. I entreated the Board to sign '*dissentient*;' but this was refused. I am therefore most anxious to make known to Your Excellency the reasons which have induced me to adopt such a measure, and with this view, I beg to enclose a copy of what I wished to accompany my signature.

Your Excellency, I trust, will do me the justice to believe, that I am not actuated by a desire to be troublesome; or that an obstinate self-will, or any over-weening conceit to be singular, is my motive:—on the contrary, I solemnly declare to Your Excellency, that I am guided by no other feeling than a conscientious adherence to what I conceive to be the truth, and I cannot help observing to Your Excellency that had the enquiry in question been conducted on more enlarged and on more philosophical principles, I humbly think the results might have been different, and more in accordance with the principles of true reasoning. Your Excellency is well aware that the laws which govern pestilence are but little known; and that it is becoming to speak doubtingly, and to speak with modesty, on a subject so very obscure, and so very open to be darkened with fallacy. I therefore hope Your Excellency will approve of my not having attached my signature to a document which I am disposed to consider somewhat faulty.

I have the honor to be, &c.

(Signed) Hugh Fraser, Surgeon.

*His Excellency Gen. Sir George Don,*
*&c. &c. &c.*

*Dissentient*—1st. Because moral testimony has been refused—the only testimony which, to my view, possibly could have been accepted to confirm the question of the liability or non-liability of an individual to a second attack of the yellow fever. Upon such testimony rests the whole of the valuable facts collected by the Board of Commissioners and the Anglo-French Commission. Had this species of testimony been received, many instances of second attack during the late epidemic might have been adduced.

2dly. Because the numerous relapses which have occurred during the epidemic fever of 1828, negative, in my opinion, the supposition that the malady bears any analogy to the order of exanthematous diseases; besides many of the relapses are of such a nature as to induce me to consider them unequivocal examples of second attack.

3dly. Because the immunity which has been observed in Gibraltar, appears to be entirely dependant on the individuals seldom changing their place of residence—very seldom the latitude. Viewing this fever, therefore, to be of malaric origin, the immunity observed, is a matter of no surprize:—it is in perfect accordance with what has been recorded of the black vomit fever of the West Indies, and America; where second, and even third attacks have been known to take place, whenever the individual lost his climatic constitution by residing a certain time in a northern latitude."

That the doctrine of non-liability to second attacks has been "fairly" brought before the public, Mr. Fraser denies. A species of evidence, he observes, is admitted as proof, when it favours the doctrine; but is refused when it has a contrary tendency.

"For example, two men come before the Board, who had been ill (we suppose during some former epidemic) with exactly the same symptoms—namely, headach, pains in the loins, sickness, and nausea, with other marks of fever, and both recover. Another epidemic takes place—one of these men escapes, the other is seized with the fever—the man who escaped, according to the mode in which this Board received evidence, would have been put down as a case of immunity; the other, as not having had the fever at any former period, and, of course, a case of first attack—only, forsooth, because the man must have been mistaken as to his first illness!! In short the *slightest indisposition* during any *former* epidemic, was put down as an attack of the fever, when the individual escaped during the *last*."

Mr. Fraser, however, does not attempt to deny that the cases of immunity have been very numerous—but the rule of immunity is far, he says, from being absolute. In papers which he is preparing for the press, "many well-authenticated cases of second attacks will be shewn."

"Without vouching for the accuracy of the 225 hospital attendants taken ill among the military, during the epidemic, according to Dr. Barry, the simple statement of facts is;—

1st. That, for the period of about one month from the commencement of the epidemic, no hospital servant was taken ill at all, though in the closest attendance on the sick.

2dly. That when the disease began to appear among them, it was not until it had begun amongst the inhabitants of the south district, in which the hospital for the military is situated.

3dly. That the first hospital servants taken ill, were the cooks and watermen, whose services did not bring them into the wards of the sick.

4thly. That, according to a nominal list (by Deputy Inspector Gillkrest, lately surgeon of the 43d Regiment) officially given in to the Board of Commissioners at their 45th sitting, it appears that of 69 men of the 43d Regiment, who, during the first month of the epidemic, were exposed in the wards day and night, in parties of from four to six each, for the space of 24 hours, the number of them attacked was found at the close of the epidemic, not to have exceeded the proportion of the general mass of the regiment attacked. Two thirds of them indeed were never attacked at all; and it was shown by that list (signed by the Adjutant of the Regiment) that those of the above 69 who were taken ill, had mounted guards in the town, and after they had been on duty in the hospital; and that the bulk of them were not taken ill till a period of from 15 days to two months, after performing the duty referred to.

5th. That it appears that in hospitals placed out of the influence of the infected atmosphere of the rock, no servant was attacked, though several were exposed, who were susceptible in the sense of not having had the disease before. This was shown to be the case, in the hospital establishments of the 73d and 94th, removed about the middle of the epidemic season to Wind Mill Hill Barracks and in the small hospitals of the 12th 42d and 43d Regiments, established near the close of the season, outside the walls, near the neutral ground.

We regret that we are unable to afford space for the table of cases, *not febrile*, admitted into the Regimental Hospital of the Royal Welsh Fusiliers, from the 1st of September till the 30th of October, shewing the number of men attacked there by the epidemic, and the length of time that must have elapsed after their exposure to the contagion (if it existed) and the coming forth of the fever.

"Taking the mean time of exposure in residence in hospital, and the interval from this mean to the date of attack, as the latent period of Bulam, the mean latent period in the eleven subjects attacked, will be, setting fractions aside, no less than 33 days! the duration of exposure of the eleven attacked, taking the mean and setting aside fractions, is 10 days, while the exposure of the 25 who escaped the contagion, is, setting aside fractions, 14 days."

The postulates 9, 10, and 11 remain to be noticed.

"Three modes of treatment—1st. Mercury with a view to salivate rapidly. 2d.

Free bleeding. 3d. Oily and other mild aperients. The first was by far the least successful, the third the most so.

10th. Some cases (7) of icterus appeared in 1829, from May to September. Three of these had no fever—four had passed the disease in other years.

11th. No atmospheric or other physical phenomena different from those of the five preceding years, were noticed in Gibraltar in 1828."

"Dr. Barry's opportunity of forming a correct estimate of the comparative merits of the different modes of treating yellow fever were so extremely limited, that any observations of his, on this head, must be received with caution. He arrived towards the decline of the disease, when it had assumed a milder type, and perhaps did not see twenty cases altogether;—nevertheless, he had the modesty to condemn the use of '*mercury*' in toto, before he had seen a single case; and took no small pains to deprecate it as the most ruinous and pernicious of medicines.

A small detachment hospital on the glacis, on the north front, was given in charge to him, and it was very soon discovered that his success was not greater than that of his neighbours, with all his boasting; for I believe that even by the aid of his own energetic treatment of a *cataplasm of port wine and bread* to the pit of the stomach, and *five leeches to the ankles,* he lost every fever-patient under his care, who had the disease in a severe form. Of this latter description of patients, however, he had but very few. None of us, it is true, can boast of much success in the treatment of the disease; those, however, of the greatest experience, are inclined to give the preference to the medicine which was so much and so loudly condemned by Dr. Barry. I myself, indeed, believe that, in the more concentrated forms of this fever, it makes very little difference what mode of treatment is adopted. The disease will run on to a fatal termination. With regard to the anatomical characters of the disease, to which I promised to advert, the Doctor's opportunities were equally limited with those he had of treating it. On arriving here he put himself under canvas, on the Neutral Ground, beyond the reach of the epidemic influence, and only had charge, as I have stated, for a short time, of the Detachment-hospitals of the 12th, 42d, and 43d Regiments, where the mortality, I believe, did not exceed eight or ten; and, to the pathological knowledge that was to be gained elsewhere at the Military and Civil Hospitals, the Doctor was a stranger, for I can assert, without the fear of contradiction, that he very seldom visited either of these establishments, so that, in truth, any facts advanced by him on these two heads, can be only from *hear-say;* and I shall take no farther notice of them."

Mr. Fraser animadverts on Dr. Barry's assertion that there were no atmospherical or other physical phenomena in 1828, different from those of the five preceding years. The average heat of the last four months of 1828 was greater, Mr. F. avers, than of the preceding years; though he pretends not to attribute the fever to this source, nor to explain why epidemics are produced in one year more than in another.

We must now close our review of Mr. Fraser's paper, reiterating our regret that men who see the same facts should so materially differ in the relation of them, and still more in the inferences which they draw from those facts. For our own parts, we greatly distrust the evidence which has been so often brought forward, in various countries, for the support of the doctrine of imported contagions. It is a most unnatural, and unfeasible doctrine, and is every day losing ground in the opinions of those best qualified to form a correct as well as an unbiassed opinion on the subject. The fact, if it be one, that an attack of the Gibraltar fever confers an immunity from subsequent seizures, is a very curious one, but we apprehend it will turn out to be nothing more than that degree of security enjoyed by those who have undergone any severe fevers either in northern or southern countries. How few do we find encounter a second attack of typhus fever in London? It would probably be extremely difficult to collect a dozen of well authenticated cases of this kind. Fevers are very different from inflammations, in this respect. The latter predispose the individual to repetitions of the disease even from slight causes—whereas the former confer a considerable power of resistance to the common causes that produce the first attack.

